# CAM-Delam: an in vivo approach to visualize and quantify the delamination and invasion capacity of human cancer cells

**DOI:** 10.1038/s41598-020-67492-7

**Published:** 2020-06-26

**Authors:** Tamilarasan K. Palaniappan, Lina Šlekienė, Anna-Karin Jonasson, Jonathan Gilthorpe, Lena Gunhaga

**Affiliations:** 1 Umeå Centre for Molecular Medicine, Umeå University, 901 87 Umeå, Sweden; 2 Department of Pharmacology and Clinical Neuroscience, Umeå University, 901 87 Umeå, Sweden

**Keywords:** Biological models, Metastasis

## Abstract

The development of metastases is the major cause of cancer related death. To develop a standardized method that define the ability of human cancer cells to degrade the basement membrane, e.g. the delamination capacity, is of importance to assess metastatic aggressiveness. We now present the in vivo CAM-Delam assay to visualize and quantify the ability of human cancer cells to delaminate and invade. The method includes seeding cancer cells on the chick chorioallantoic membrane (CAM), followed by the evaluation of cancer-induced delamination and potential invasion within hours to a few days. By testing a range of human cancer cell lines in the CAM-Delam assay, our results show that the delamination capacity can be divided into four categories and used to quantify metastatic aggressiveness. Our results emphasize the usefulness of this assay for quantifying delamination capacity as a measurement of metastatic aggressiveness, and in unraveling the molecular mechanisms that regulate delamination, invasion, formation of micro-metastases and modulations of the tumor microenvironment. This method will be useful in both the preclinical and clinical characterization of tumor biopsies, and in the validation of compounds that may improve survival in metastatic cancer.

## Introduction

It is estimated that metastases, i.e. cancer cells that spread from their site of origin to distant organs and initiate the formation of secondary tumors, are responsible for around 90% of mortality in cancer patients^1^. Hence, metastasis is both a critical measure of cancer severity and an important target for cancer therapy. The formation of cancer metastases is a complex process, which includes epithelial-to-mesenchymal transition (EMT) as one of several key steps. EMT is a normal cellular process activated in invasive and metastatic cancer cells, in which adherent epithelial cells loses their polarity and gain a more loosely oriented and invasive mesenchymal phenotype^2,3^. Delamination, defined as the degradation of the basal lamina and basement membrane, facilitates the transmigration and spread of cancer cells to other tissues and organs^4,5^.

Clinical methods to detect cancer invasion and metastases include tomography, magnetic resonance imaging (MRI) and pathological examination of patient biopsies. However, metastatic cancer colonies are often too small to detect at the time the primary tumor is observed and treated^6^. Thus, there is a need for methods to define the metastatic aggressiveness of a primary tumor as early as possible, which would help to support medical approaches to identify and treat cancer at an early stage.

The chick chorioallantoic membrane (CAM) is formed after embryonic day (E) 5 by partial fusion of the chorion and the allantois, and by E12 surrounds the whole embryo^7^. The CAM is primarily composed of type IV collagen and laminin, which mimics the basement membranes in humans^8,9^. The CAM has a rich vascular system and acts as an organ for gas-exchange during chick embryonic development. The CAM has been exploited as a relatively simple, time- and cost-efficient model to study various cancer related processes including angiogenesis, cancer cell invasion, metastasis formation and tumor progression^10–13^. One important aspect of this in vivo system is that the chick embryo is immunodeficient up until E18, just prior to hatching^14^, affording the possibility to transplant donor cells and tissue without an adverse host response. In addition, the chick in vivo system is relatively unhindered by ethical requirements prior to E14. However, a significant challenge using CAM studies as with other chick in vivo assays that include opening of the egg shell, has been the general low (10–50%) survival^15–19^.

Laminins are a family of secreted αβγ heterotrimeric glycoproteins that self-assemble into a cell-associated network in all basement membranes, as well as the CAM^20,21^. Factors that are up-regulated during metastases, including matrix metalloproteinases (MMPs), membrane-type MMPs (MT-MMPs), ADAM (a-disintergin and metalloproteinase) and ADAMTS (ADAM with thrombospondin motifs), degrade macromolecular components such as laminin to facilitate tumor cell invasion and metastasis^22,23^. Thus, the development of in vivo methods to study delamination, which proceeds metastasis formation, is useful in order to estimate the ability of cancer cells to cause metastases, and also to screen for anti-delamination/anti-metastatic compounds.

Here, we present a convenient solution to overcome the low survival rate of chick embryos in CAM assays, and demonstrate the use of an internal humidity chamber that increases survival up to 80–90%. Most importantly, we present the CAM-Delam model as an innovative phenotypic technique that can be used to visualize and quantify the changes from intact to damaged basal lamina within a period of hours to a few days. The use of immunohistochemistry facilitates the scoring of the capacity of various cancer cells to degrade the basal lamina. In addition, we demonstrate that the CAM-Delam assay can be used as a model to study the molecular mechanisms that regulate delamination, invasion, the formation of micro-metastases and modulation of the tumor microenvironment.

## Results

### An internal humidified chamber increases embryo survival in CAM assays

To develop an in vivo model to determine the delamination capacity of cancer cells, we turned to the chick chorioallantoic membrane (CAM) as a model system. The first challenge was to improve the low survival of chick embryos using previous incubation protocols of CAM-assays^15,17–19^, in which chick embryos at incubation Day 3 were transferred to a Petri dish or similar for continued incubation until Day 13.

**Figure 1:**
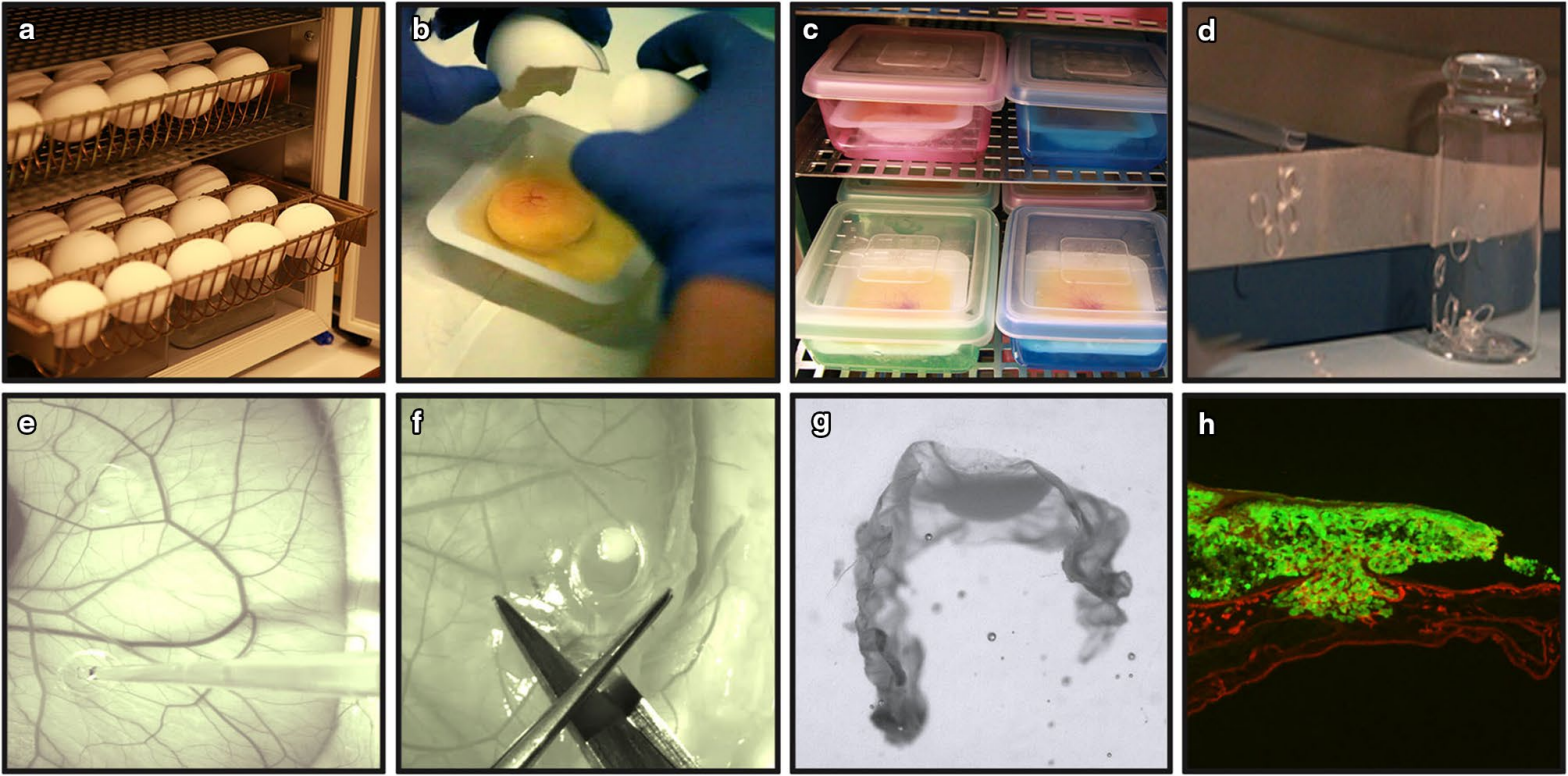
Overview of the CAM-Delam assay. (**a**) Fertilized chick eggs were incubated horizontally. (**b**) On Day 3 of incubation, eggs were cracked and laid in a weighing boat in (**c**) an internal humidified chamber. (**d**) Silicon ring preparation. (**e**) On Day 10 of incubation, 1 × 10^6^ cells were seeded on the CAM using a pipette. (**f**) At different time points, the CAM membrane with the growing cancer cells were dissected out, fixed, sucrose treated and (**g**) positioned in frozen section medium. (**h**) Sectioned CAM with associated GFP^+^ cancer cells (green), followed by anti-Laminin immunohistochemistry (red).

Fertilized chick eggs were incubated horizontally (Day 0) without rotation (Fig. 1a). On Day 3 of incubation, eggs were cracked and the yolk and its associated embryo were quickly laid into a weighing boat (Fig. 1b and Video S1). Embryos with a beating heart and an intact underlying yolk were considered as healthy, whilst eggs with a leaking yolk, damaged blood vessels or an unfertilized appearance were discarded. Eggs with a healthy appearance were transferred in their weighing boat to an internally humidified chamber, made up by a plastic box containing 50 ml sterilized H_2_O and closed with a transparent lid (Fig. 1c and Video S1). The transparent lid allowed for daily observations of embryonic development without the need to open the internal humidified chamber, which minimized infection and improved survival.

Embryos were maintained in humidified chambers until Day 10–13 of incubation and this resulted in an average survival of 89–81% on Day 10 and Day 13 of incubation, respectively (Fig. S1a-d). In contrast, conducting the incubation in Petri dishes alone, resulted in 40% embryo survival on Day 10, which dropped to 11% on Day 13 (Fig. S1a,b). When culturing in weighing boats, but without an internally humidified chamber, 49% of the embryos survived on Day 10 and 18% on Day 13 (Fig. S1c,d). Thus, by the use of weighing boats and an internally humidified chamber, we were able to significantly improve embryonic survival during long-term embryo culture for CAM-assays.

### The establishment of the CAM-Delam assay and CAM-Delam scoring

Next, we used the optimized method to culture embryos until Day 10 of incubation. At this point, sterilized silicon rings with an inner diameter of 4 mm were placed on the top of the CAM to restrict the area for cell seeding, but never placed on the largest blood vessels (Fig.1d, e and Video S2). Up to nine rings can be used in the same embryo, which cover most of the CAM, without compromising the chick survival. Human cancer cells (1 × 10^6^ cells suspended in 20 µl collagen/RPMI-mix) were seeded in each silicon ring (Fig. 1e and Video S2) and incubated for another 14–86 h (h). At different time points, the CAM membrane with the growing cancer cells was dissected out (Fig. 1f), fixed, cryo-protected in sucrose, embedded in cryo-sectioning medium (Fig. 1g) and stored at -80ºC until further analyses.

**Figure 2:**
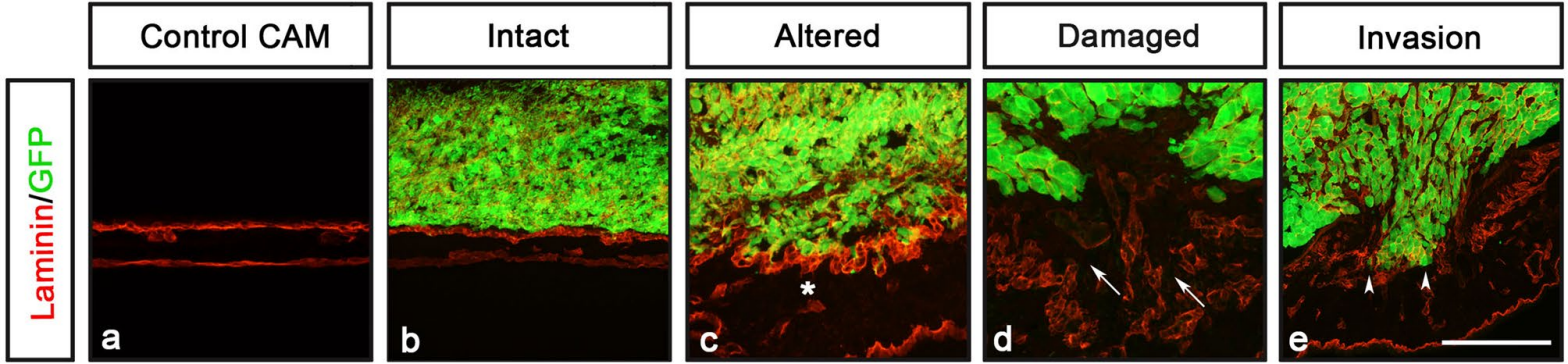
CAM-Delam scoring. (**a**–**e**) An example of how CAM-Delam results are scored in relation to the basal lamina of the CAM, here visualized by anti-Laminin (red), and cancer cells that express GFP (green). (**a**) Non-affected control CAM. (**b**–**e**) The capacity of cancer cells to degrade the basal lamina and invade can be scored into four categories; (**b**) intact Laminin, (**c**) altered, but not damaged Laminin (indicated by asterisk), (**d**) damaged Laminin without cell invasion (indicated by arrows), (**e**) damaged Laminin with cell invasion (indicated by arrowheads). Scale bar: 100 μm (**a**–**e**).

Frozen CAM-samples were cryo-sectioned at 10 µm and sections were placed onto consecutive slides. After immunohistochemistry, stained sections were analyzed for the morphology of the basal lamina, cancer cell invasion (defined as human cancer cells that have crossed the chick basal lamina layer into the chick mesodermal layer), CAM thickening and vessel formation (Fig. 1h). Our results showed that the capacity of cancer cells to degrade basal lamina and invade the mesenchyme could be scored into one of four categories; (1) intact basal lamina without visible alterations, (2) altered, but undamaged basal lamina, (3) damaged basal lamina without cell invasion, (4) damaged basal lamina with cell invasion (Fig. 2).

### The CAM-Delam assay visualize the metastatic capacity of different cancer cell lines

**Figure 3:**
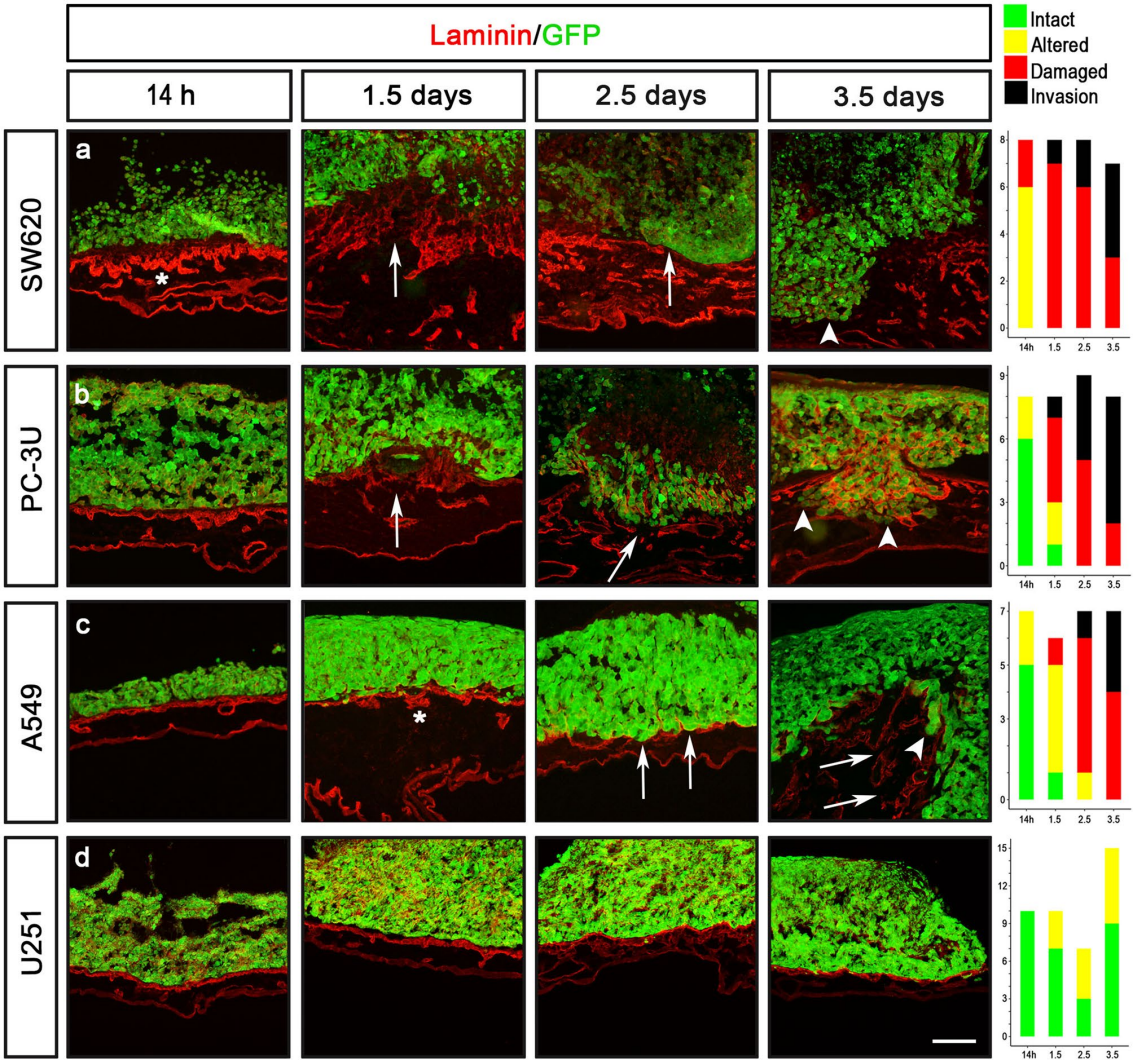
Demonstrated delamination capacity of different cancer cell lines using the CAM-Delam assay. The prostate (PC-3U), colon (SW620), lung (A549) and glioblastoma (U251) cells were seeded on the CAM, following a period of incubation from 14 h to 3.5 d. (**a**) SW620 cells induced major alteration of Laminin after 14 h, and delamination and cell invasion after 1.5 d. (**b**) PC-3U cells induced minor alteration of Laminin after 14 h, damaged Laminin after 1.5 d, and invasion after 2.5 d (**c**) A549 cells induced minor alteration of Laminin after 14 h, damaged Laminin after 2.5 d, and cell invasion at 3.5 d. (**d**) U251 cells induced minor alteration of Laminin after 1.5–3.5 d. The results of CAM-Delam scoring are shown in the right panel. The y-axis indicates number of samples, and the x-axis indicates time points of culture. Asterisk indicate altered Laminin distribution; arrows indicate damaged Laminin, and arrowheads indicate cell invasion. Scale bar: 100 μm (**a**–**d**).

To monitor the position and morphology of human cancer cells in the CAM-Delam assay, four different cancer cell lines; colon (SW620), prostate (PC-3U), lung (A549) and glioblastoma (U251), were engineered using the CRISPR/Cas9 technique^24,25^ to carry a GFP expressing transgene in the AAVS1 locus (Fig. S2). To evaluate the established CAM-Delam assay, the four GFP expressing cancer cell lines (SW620-*GFP*, PC-3U-*GFP*, A549-*GFP* and U251-*GFP*) were used. The morphology of the CAM basal lamina, using anti-Laminin immunohistochemistry, and cancer cell invasion, using GFP expression, were analyzed at four different time points; 14 h, 38 h (1.5 days), 62 h (2.5 days) and 86 h (3.5 days). Different cell lines showed different phenotypes. SW620 cells induced major alterations in Laminin distribution by 14 h, and delamination and cell invasion after 1.5 days (Fig. 3a). PC-3U cells induced minor alterations in Laminin distribution by 14 h, and damaged Laminin after 1.5 days, which increased after 2.5 days in combination with cell invasion (Fig. 3b). A549 cells induced minor alterations in Laminin by 14 h, which increased at 1.5 days with clear damage to Laminin after 2.5 days, which at 3.5 days was detected in combination with cell invasion (Fig. 3c). In contrast, U251 cells only induced minor alterations of Laminin by 1.5–3.5 days, but never caused any visible damage to Laminin (Fig. 3d), and even after 5 days of culture no clear damage or invasion was observed (Fig. S3). These results suggest that SW620 cells exhibit a high delamination capacity and an aggressive invasion phenotype, followed by PC-3U and A459 cells that also show strong capacities to induce delamination and invasion, whereas U251 do not exhibit any delamination capacity and subsequently does not invade the CAM. The conclusion is that the CAM-Delam assay can be used to visualize and quantify the delamination and invasion capacity of human cancer cells.

Another phenotype that could be observed in the CAM-Delam assay was the thickening of the chick mesenchymal layer after 1.5 days in response to the seeded cancer cell lines that exhibited metastatic potential; PC-3U, SW620 and A549 cells (Fig. 3a–c and Fig. S4b-d). Moreover, an increase in blood vessel formation, indicated by anti-von Willebrand Factor immunohistochemistry staining, was observed (Fig. S4g-i). In contrast, thickening of the mesenchyme or increased vessel formation were not observed in response to the non-metastatic U251 cells, or in the control CAM without any seeded cells (Fig. S4a, e, f, j). Together this shows that metastatic cancer cells induce a visible response in the surrounding host cells over a period ranging from of a few hours to days.

Next, we tested whether different cancer cells can be analyzed on the same CAM. To achieve this, we cultured (i) the metastatic cancer cells (SW620, PC-3U or A549) and (ii) the non-metastatic U251 cells in different rings, but on the same CAM for 3.5 days. Under these conditions, the delamination scoring results for each cell line remained the same, regardless of whether the lines were cultured on separate or on the same CAM (Fig. S5 and data not shown). This demonstrates that the CAM-Delam assay can be used to score the capacity of specific human cancer cell lines to degrade the basal lamina and invade, individually on the same CAM, which is a prerequisite for later invasion and metastasis formation. This points to a good assay throughput and reproducibility, with the option to examine potential inhibitors and control conditions on the same CAM.

### Assessing the potential of metastatic versus non-metastatic organ specific cancer cells using the CAM-Delam assay

To evaluate diagnostic potential of the CAM-Delam assay, we conducted a blinded comparison between previously described non-metastatic (HOS) and metastatic (143B) osteosarcoma (OS) cancer cell lines^26^, labelled as #OS1 and #OS2. The #OS1 and #OS2 cells were analyzed in the CAM-Delam assay at four different time points; 14 h, 1.5 days, 2.5 days and 3.5 days. Both #OS1 and #OS2 were found to express Laminin, which prohibited clear visualization of the CAM basal lamina when detected with anti-Laminin (Fig. S6). To overcome this issue, we instead used E-cadherin to detect Laminin-producing epithelial cells of the chick CAM (Fig. S7a-c). Notably, the laminin layer was found to be intact, even when the E-cadherin^+^ epithelia was disrupted (Fig. S7d-i), demonstrating that disrupted E-cadherin^+^ epithelia together with the presence of invasive cells, indicated delamination and invasion properties of cancer cells.

**Figure 4:**
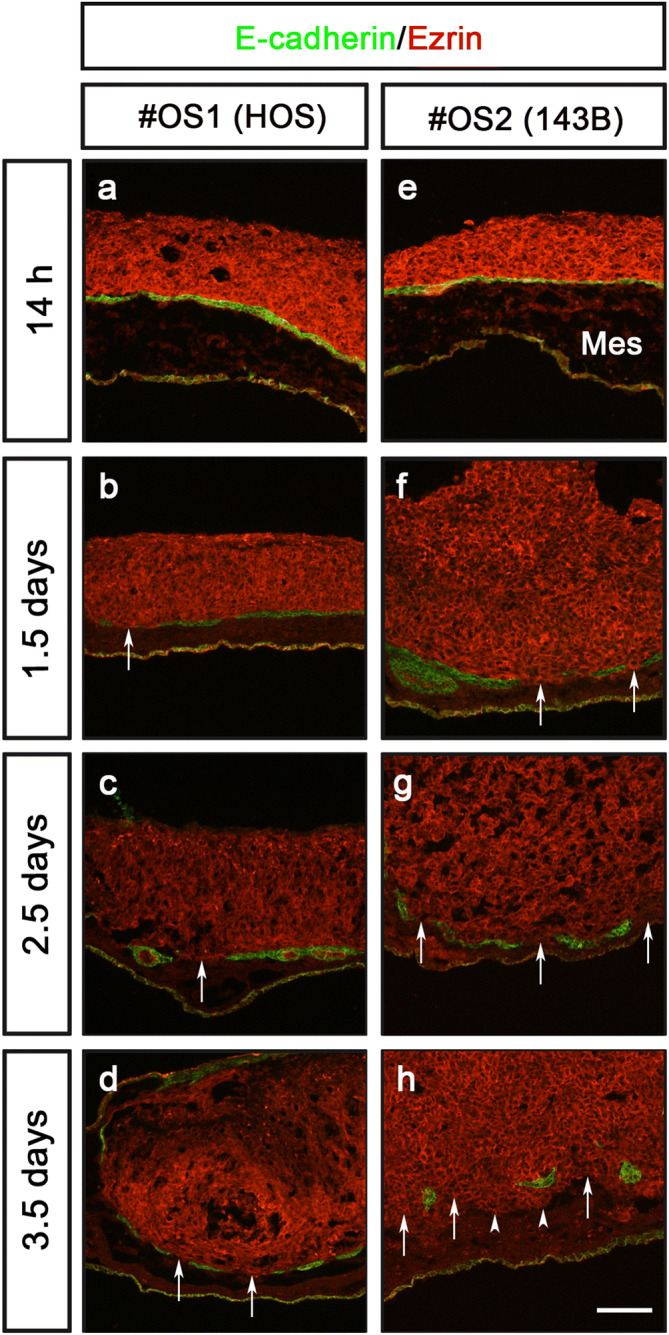
Assessing the potential of metastatic versus non-metastatic organ specific cancer cells using the CAM-Delam assay. Results of a blinded test of two different osteosarcoma cell lines #OS1 (HOS) and #OS2 (143B) analyzed at 14 h, 1.5 d, 2.5 d and 3.5 d. n = 4 per cell line and time point. (**a**-**h**) Cancer cells are observed by Ezrin (red) and the location of Laminin-producing cells in the CAM by E-Cadherin (green). (**a**–**d**) Using the #OS1 (HOS) cells, minor disruption of E-cadherin was detected at 1.5–3.5 d (arrows in **b**–**d**), but no invasion into the mesenchyme was observed. (**e**–**h**) The #OS2 (143B) cells promoted increased disruption of the E-cadherin epithelia (arrows in **f**–**h**) and cancer cell invasion into the mesenchyme (arrowheads in h) from 1.5–3.5 d. (**f**–**h**) An increased growth of the #OS2 (143B) cell line into a tumor-like formation on CAM was observed during 1.5–3.5 d. Abbreviations: Mes – mesenchyme. Scale bar: 100 µm (**a**–**h**).

Neither the #OS1 or #OS2 cells induced a disruption in the E-cadherin staining after 14 h (Fig. 4a, e). However, at 1.5–3.5 days, minor disruptions of the E-cadherin^+^ epithelia without invasion of the mesenchyme was observed in #OS1 seeded CAMs (Fig. 4b–d). In contrast, #OS2 cells caused increased disruption of the E-cadherin^+^ epithelia from 1.5 to 3.5 days, together with increased growth of the #OS2 cells into tumor-like clusters on the CAMs (Fig. 4f–h). Moreover, after 3.5 days, cell invasion into the mesenchyme was evident in #OS2 seeded CAMs (Fig. 4h). This indicated that #OS1 were the non-metastatic HOS cells, and #OS2 were the metastatic 143B OS cell line. These conclusions were subsequently confirmed by the provider of the blinded OS cell line (Emma Persson, Dept. of Radiation Sciences, Umeå University). Thus, these results indicate that the CAM-Delam assay is a valuable model with which to screen for the delamination capacity and metastatic aggressiveness of cancer cells.

### Analyzing factor-induced delamination of U251 cells using the CAM-Delam assay

Hypoxia is an important factor in the development of many cancers and is involved in the induction of metastasis, in which hypoxia-inducible factor-1α (HIF1-α) and HIF-1β play key roles by upregulating several pro-oncogenic factors^27,28^. CoCl_2_ is a known chemical inducer of HIF1-α and HIF-1β, which in turn promotes vascular growth and EMT^29^. To examine whether CoCl_2_ could induce delamination capacity in a non-metastatic cell line, U251 cells were pre-treated with 200 µM CoCl_2_ for 24 h, washed thoroughly in PBS before being used in the CAM-Delam assay.

**Figure 5:**
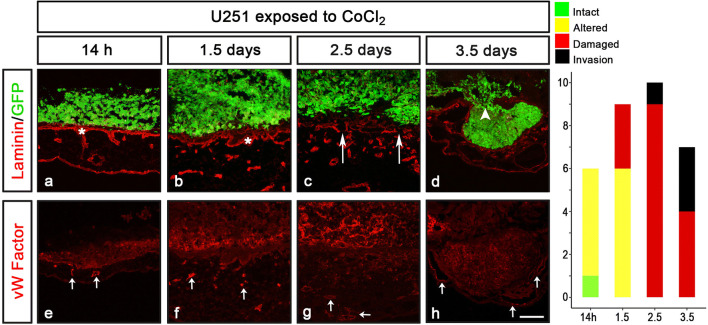
U251 induced delamination in response to CoCl_2_ exposure. (**a**–**h**) U251 cells pre-exposed to 200 µM CoCl_2_ prior to cell seeding on the CAM and further culture. (**a**–**d**) In response to CoCl_2_ exposure, U251 cells induced altered Laminin by 14 h, and clear damage of Laminin after 2.5 d, followed by invasion at 3.5 d. (**e**–**h**) Blood vessel recruitment indicated by anti-von Willebrand Factor (VW Factor) immunohistochemistry was detected after 14 h and increased at 1.5 d. Von Willebrand Factor staining was also detected in U251 cells close to the CAM. The results of CAM-Delam scoring are shown in the right panel. The y-axis indicates number of samples, and the x-axis indicates time points of culture. Asterisk indicate altered Laminin distribution; long arrows (**c**) indicate damaged Laminin; arrowhead indicate cell invasion; short arrows (**e**–**h**) indicate blood vessels. Scale bar: 100 μm (**a**-**h**).

In contrast to un-treated U251 cells (Fig. 3d), the CoCl_2_ pre-treated U251 cells triggered alteration of Laminin after 14 h, damage by 2.5 days and invasion of cells by 3.5 days (Fig. 5a–d). In addition, already by 14 h, the CoCl_2_ pre-treated U251 cells induced thickening of, and recruitment of blood vessels within, the chick mesenchymal layer (Fig. 5e–h). These phenotypes were never observed after seeding untreated U251 cells (Fig. 3d and S4e, j). Thus, induction of delamination in non-invasive U251 cells may be used in CAM-Delam assay to understand factor-induced delamination.

### Examining inhibition of delamination using the CAM-Delam assay

**Figure 6:**
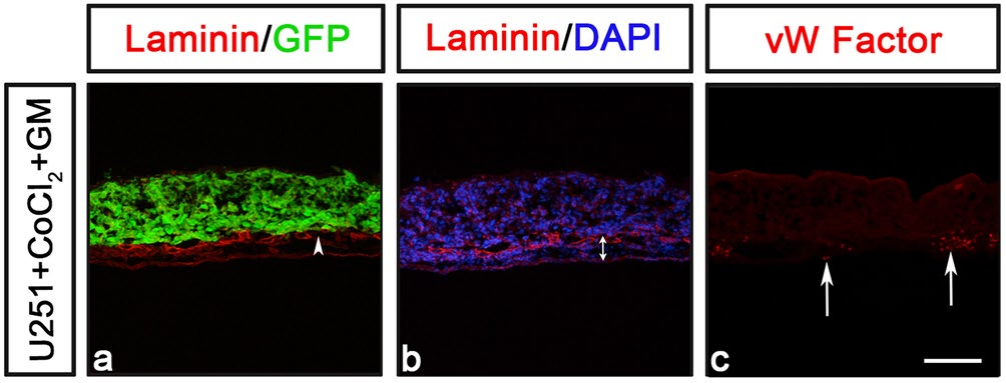
Examining inhibition of delamination using the CAM-Delam assay. (**a**, **b**) U251 cells pre-treated with the broad MMP inhibitor GM6001 (for 1 h) and then together with CoCl_2_ (for 24 h) before washing and seeding on the CAM and cultured to 3.5 d. (**a**) Minor damage of the Laminin, but no invasion was detected (**a**; n = 3). (**b**, **c**) No thickening of the mesenchyme (**b**; n = 7), or increased blood vessel formation (**c**; n = 3) was observed. (**a**–**c**) Arrowhead in (**a**) indicate minor damage of Laminin, double arrow in (**b**) indicate the mesenchyme thickness, and arrows in (**c**) indicate blood vessels. Scale bar: 100 μm (**a**-**c**).

Next, we examined whether the CAM-Delam assay can be used to study inhibition of the delamination process. To assess this, U251 cells were first exposed to 25 µM of the broad MMP inhibitor GM6001 1 h, before pre-treatment with 200 µM CoCl_2_ as described above. After a total 25 h of GM6001 exposure, the cells were washed thoroughly in PBS before being assayed by CAM-Delam for 3.5 d. GM6001 treatment clearly suppressed the delamination and invasion capacity of CoCl_2_ pre-treated U251 cells cultured for 3.5 d (Fig. 6a). Moreover, at 1.5–3.5 d, GM6001 treatment resulted in suppressed thickening of the mesenchyme and reduced blood vessel formation compared to the CoCl_2_ exposure (Fig. 6b, c and data not shown). These results provide evidence that the CAM-Delam assay can be used to screen for new delamination and invasion inhibitors, and to define molecular pathways that regulate delamination in cancer.

## Discussion

Here we describe the CAM-Delam assay as an innovative and flexible approach to evaluate the metastatic capacity and aggressiveness of human cancer cells, which can be determined by scoring basal lamina alterations and delamination within a period of hours to a few days. One important innovation is the use of an internally humidified chamber during embryo incubation, which led to a survival increase from 10–50% to 80–90%, resolving previous technical problems due to low survival rates in various CAM assays. We also show that the CAM-Delam approach can be modified to accommodate different types of functional assays in order to address the molecular and cellular mechanisms that underlie delamination, invasion, micro-metastasis formation and modulations of the tumor microenvironment, by studying induction and inhibition of these processes. Challenges with the CAM-Delam method might be to examine non-GFP cancer cell lines, which requires relevant antibodies to identify the investigated cancer cells, and to score cancer cell lines that express high levels of laminin, which can, but does not have to be a disadvantage, to determine morphological changes of the basal lamina.

The survival of chick embryos in various CAM assays has been reported to be around 10–30%^15,17–19^. The work by Dohle et al., emphasizing the importance of cleaning the egg shell to minimize infection and cracking eggs at Day 3 of incubation to improve survival, only attained ~ 50% survival^16^. In comparison, *in ovo* CAM assays have a relatively high rate of embryo survival (75–90%), while also having limitations due to problems with membrane drying and also because it is difficult to access and visualize the CAM *in ovo*^30–32^. By the use of an internally humidified chamber, we found that survival of in vivo incubated chick embryos was reliably attained at 80–90%, which is a significant improvement from previous methods. This method of culture would be not only beneficial in different types of CAM-assay, but also in a range of other in vivo*/ex ovo* chick experiments with a need to access the CAM or the embryo whilst maximizing survival.

Using the CAM-Delam assay to monitor local disruptions of the basal lamina, we were able to determine the metastatic capacity and aggressiveness of human cancer cells in vivo within 0.5–3.5 days. This disruption of the basal lamina is a prerequisite for the later formation of microtumors and organ metastases, in both proximal and distal locations, that previous CAM methods have analyzed, and that requires 5–10 days of culture^33–35^. Thus, the relatively fast-delivered results from the CAM-Delam assay are facilitated by the scoring of local alteration and degradation of the basal lamina, which also indicates the position of the delamination with no need to monitor metastasis formation. Most CAM assays are time efficient compared to mammalian grafted-tumor models, which requires a minimum of 4–6 weeks before metastasis formation can be analyzed^36,37^. By testing four well characterized cancer cell lines from various, clinically important cancer types, our results show that the delamination capacity of cancer cells can be divided into at least four categories; (1) intact basal lamina without visible alterations, (2) altered, but undamaged basal lamina, (3) damaged basal lamina without cell invasion, (4) damaged basal lamina with cell invasion. Three cancer cell lines; prostate (PC-3U), lung (A549) and colon (SW620) cancer cells, underwent clear delamination, but to different extents over time, whereas glioblastoma (U251) cells did not induce delamination. Our CAM-Delam scoring results are in line with previous in vivo mouse metastatic models that have characterized high metastatic capacity of PC-3U, A549, SW620, and 143B cell lines^38–41^, and non-metastatic capacity of U251 and HOS cell lines^40,42^, supporting the predictive value of the CAM-Delam method. Thus, within only a few days the CAM-Delam assay can give an estimation of metastatic potential of cancer cells. Moreover, our observation of thickening of the chick mesenchyme and increased blood vessel formation within after contact with metastatic cancer cells, suggest that the CAM-Delam assay may be used to study how cancer cells affect the tumor microenvironment.

Staging of cancer is a commonly used clinical method to determine the severity of a cancer type. The TNM staging system has been the main method used for cancer reporting for several decades^43,44^. TNM staging accounts for tumor size (T), whether the cancer has spread to the lymph nodes (N) and distant metastasis (M). Accurate information on the severity of a cancer around the time of diagnosis is an important component of cancer care, defining disease prognosis and in delivering the most effective treatment. In addition to the TNM staging, the CAM-Delam model has the potential to be a useful complement assay to provide rapid quantification about the metastatic capacity of individual cancer types, perhaps even prior to metastasis formation. To assess this, analysis of various cancer biopsies using the CAM-Delam assay is required, in combination with clinical follow up of patient outcome.

There is a clear association between increased levels of HIF-1/2α proteins and increased resistance to radiotherapy and chemotherapy, cancer progression and patient mortality^27^. Therefore, it is important to better understand how HIF-1/2α proteins promotes cancer. Our results demonstrate that the CAM-Delam assay can not only be used to identify molecular mechanisms that play a role in triggering delamination, like HIFs, but also to examine the activity of anti-delaminating/anti-metastatic compounds. It is important to note that most mammalian models are not suitable for investigating delamination, since tumor cells are injected or grafted into the blood system and/or connective tissue. Therefore, these approaches physically bypass the basement membrane of the surface epithelium, which is the primary barrier to cancer cell invasion^45–47^.

## Methods

All methods were carried out in accordance with relevant guidelines and regulations.

### CAM-Delam assay

#### Egg incubation and breakage

Fertilized white Lohman and Bovan chick eggs were obtained from Strömbäcks Ägg, Vännäs, Sweden. The use of fertilized chick eggs prior to E14 does not require an ethical permission. The chick eggs were incubated horizontally (day 0) in an egg incubator (Fiem) without rotation in relative humidity of 70% and temperature of 37.5 °C. On Day 3 of incubation the eggs were sprayed with 70% ethanol and left to dry at room temperature (RT). Egg was cracked and the yolk and its attached embryo were transferred to a weighing boat (VWR International). The weighing boat was placed in a 0.4 L plastic box (Esclain) containing 50 ml sterilized H_2_O and closed with a transparent lid, creating an internally humidified chamber, in which embryos were further incubated.

#### Preparation of human cancer cell lines for the CAM-Delam assay

The human cancer cell lines (U251-*GFP*, SW620-*GFP*, PC-3U-*GFP*, and A549-*GFP*; for generation, see below) were washed with 1xPBS, trypsinized (0.05%; Gibco), resuspended in RPMI medium and counted by the Trypan Blue (Invitrogen) exclusion method in a cell counter (Countess II FL Automated Cell Counter, Invitrogen). 50 × 10^6^ cells were centrifuged (Rotanta 480 R, Hettich zentrifugen) at 500 RCF for 5 min. The supernatant was discarded and the cell pellet was mixed with 1 ml collagen/RPMI-mix (ratio 1:3), containing 250 μl of type I collagen (PureColEZ Gel, Advanced Biomatrix) and 750 μl of RPMI medium supplemented with 10% Fetal bovine serum (FBS; Life Technologies) and 1% (v/v) penicillin–streptomycin (PS; Gibco). Cell suspensions were stored on ice prior to seeding in CAM-Delam assays.

#### Silicon ring preparation and seeding of cancer cells on CAM

Silicon rings of 1 mm thickness were prepared by cutting a silicone tube with an inner and outer diameter of 4 and 5 mm, respectively (VWR). Silicone rings were placed in a glass chamber, sealed and autoclaved until used on the CAM. On Day 10 of incubation, up to six sterile silicone rings were placed on one CAM. 1 × 10^6^ human cancer cells dissolved in 20 µl collagen /RPMI-mix were seeded in the center of each ring. Boxes were sealed immediately and incubation was continued.

#### Isolation of the CAM and associated cancer cells, freezing and sectioning

The CAM with attached cancer cells (CAM samples) were dissected out after 14 h, 1.5 days, 2.5 days and 3.5 days after cancer cell seeding, fixed in 4% paraformaldehyde (Sigma-Aldrich) for 1 h at 4 °C, and transferred to 25% sucrose solution for 1 h at 4 °C. The silicon rings were removed from the CAM and the CAM samples were positioned in tissue embedding molds (Polysciences) in frozen section medium (NEG-50, Thermo Fisher Scientific). CAM samples were frozen and stored at − 80 °C until they were cryo-sectioned at 10 µm (HM 505 E, Microm) on consecutive microscope slides (Superfrost Plus, Menzel-Glaser).

### Immunohistochemistry

Immunohistochemistry was performed using standard protocols^48^. Briefly, sections were blocked in 10% FBS prior to primary antibody incubation at 4 °C overnight. Primary anti-rabbit antibodies used were; anti-Ezrin (Santa Cruz sc-20773; 1:100), anti-Laminin-111 (Sigma-Aldrich L9393; 1:400), anti-von Willebrand Factor (DAKO P0226; 1:100) and primary anti-mouse antibody used were anti-E-Cadherin (DSHB #7D6, 1:50). Secondary antibodies used were; anti-rabbit Cy3 (1:400, Jackson Immuno Research), anti-mouse Alexa Fluor 488 (1:400, Invitrogen). Nuclei were detected using DAPI (1:400; Sigma-Aldrich). Sections were mounted with fluorescence mounting medium (Allent Technologies).

### Scoring of delamination

Stained sections were photographed using an epifluorescence microscope (Nikon Eclipse, E800) equipped with a digital camera (Nikon DS-Ri1). The capacity of cancer cells to degrade the basal lamina and invade into the mesenchyme was scored by three individuals, in which two persons scored in a blinded manner, and the results were grouped according to four categories; (1) intact basal lamina without visible alterations, (2) altered, but undamaged basal lamina, (3) damaged basal lamina without cell invasion, (4) damaged basal lamina with cell invasion. Measurements were taken from distinct samples. The scoring results presented in graphs were generated with ggplot2;^49^ package using R software version 3.6.0.

### Design and cloning of vectors for CRISPR/Cas9n targeting of the AAVS1 locus

Guide sequences to target the *AAVS1* locus were designed using the Optimized CRISPR Design Tool (https://crispr.mit.edu25). Complimentary oligonucleotides were cloned into the Bbs*I* site of the pSpCas9n(BB)-2A-Puro (PX462) vector (Addgene plasmid # 48,141;^50^), expressing Cas9n, and confirmed by plasmid DNA sequencing. The efficiency of different pSpCas9n-sgRNA constructs was evaluated using the Traffic Light Reporter (TLR) system, in which the *AAVS1* target sequence was cloned into pCVL Traffic Light Reporter 1.1 (Sce target) Ef1a Puro (Addgene plasmid # 31,482;^51^) and used to generate a HEK293-TLR-AAVS1 stable cell line via lentiviral transduction. The selected pair of target sequence for sgRNA Target 1 was 5´-GAGCCACATTAACCGGCCCT-3´ (reverse strand), with corresponding forward and reverse oligonucleotides 5´-caccGAGCCACATTAACCGGCCCT-3´ and 5´-aaacAGGGCCGGTTAATGTGGCTC-3´. Target sequence for sgRNA Target 2 was 5´-GACCCCACAGTGGGGCCACTA-3´ (forward strand), with corresponding forward and reverse oligonucleotides 5´-caccgACCCCACAGTGGGGCCACTA-3´ and 5´-aaacTAGTGGCCCCACTGTGGGGTC-3´ (see Fig. S2a).

The sgRNA target sequences within the left homology arm of the AAVS1-CAG-hrGFP donor vector (Addgene plasmid # 52,344;^52^), as well as the puromycin cassette, were removed by linearizing with *Spe*I, blunting with T4 DNA polymerase, followed by digestion with *Sma*I and re-ligation. The final AAVS1-CAG-hrGFPΔtargetΔpuro vector contained the left and the right arm sequence of the AAVS1 locus with a CAG promotor driven hrGFP.

### Generation of GFP stable cancer cell lines

Three vectors (pSpCas9n-PX462-target1, pSpCas9n-PX462-target2, AAVS1-CAG-hrGFPΔtargetΔpuro) were introduced into the cancer cell lines; A549, PC-3U, SW620 and U251, by electroporation using a Gene Pulser Xcell Electroporation system (BIO-RAD). After plating at low density, GFP-expressing clones were selected manually using discs of filter paper soaked in trypsin–EDTA solution^53^. Genomic DNA from isolated GFP^+^ clones was extracted using the Quiagen DNA Isolation Kit. The presence of the reporter construct in the *AAVS1* locus was determined by PCR amplification using a forward primer located upstream of the left homology arm (5´-TCTCTCTCCTGAGTCCGGAC-3´) and a reverse primer located in the CMV enhancer of the CAG promoter (5´-CGGGCCATTTACCGTAAGTT-3´).

### Cell culture

The human lung cancer A549, colon cancer SW620, osteosarcoma cancer HOS and 143B cell lines were purchased from the American Type Culture Collection (ATCC). The human prostate cancer PC-3U cells represents a clone from the original PC-3 cell line (ATCC CRL-1435)^54^. The human glioblastoma U251 MG (formerly U-373 MG) originated from the European Collection of Authenticated Cell Cultures (ECACC). The four cancer cell lines stably expressing GFP; U251-*GFP*, SW620-*GFP*, PC-3U-*GFP*, and A549-*GFP*, were cultured in RPMI media supplemented with 10% (v/v) FBS, and 1% (v/v) PS (Gibco). Cells were cultured at 37 °C in the presence of 5% (v/v) CO2. Cancer cells were maintained under passage 15. Mycoplasma test (Eurofins GATC Biotech) confirmed that the cancer cells were free from mycoplasma infection, and the identity of the cancer cell lines was verified by STR profiling (Microsynth).

### Functional experiments using the CAM-Delam assay

#### CoCl_2_ and GM6001 exposure

U251 cells were pre-treated with 200 µM CoCl_2_ (Sigma-Aldrich) in RPMI-medium without serum for 24 h in culture, followed by two washes in PBS before being used in the CAM-Delam assay. To inhibit matrix metalloproteases, 25 µM GM6001 (Sigma-Aldrich) was added to U251 cells 1 h before pre-treatment with 200 µM CoCl_2_ as described above.

### Statistical analysis

Results are expressed as ± SD. Statistical significance was tested with Graph Pad version 8.3.0. using unpaired two-tailed t-test. *p* < 0.05 was considered significant (*p < 0.05; **p < 0.005; ***p < 0.0001).

## Supplementary information

Supplementary information

Supplementary Video S1

Supplementary Video S2
