# Supplementary material for: CAM-Delam: an in vivo approach to visualize and quantify the delamination and invasion capacity of human cancer cells
Source: Sci Rep. 2020 Jun 26;10:10472. doi: 10.1038/s41598-020-67492-7 (PMC7320147; doi:10.1038/s41598-020-67492-7)
Supplement: Supplementary file 1 — Supplementary information [file 41598_2020_67492_MOESM1_ESM.docx]

**Supplementary material for**

**CAM-Delam: an *in vivo* approach to visualize and quantify the**

**delamination and invasion capacity of human cancer cells**

Including:

Supplementary Figures 1-7

Supplementary Video Captions 1-2

by

Tamilarasan K. Palaniappan^1^*, Lina Šlekienė^1^, Anna-Karin Jonasson^2^, Jonathan Gilthorpe^2^ and Lena Gunhaga^1^*

^1^Umeå Centre for Molecular Medicine, Umeå University, Umeå 901 87, Sweden

^2^Department of Pharmacology and Clinical Neuroscience, Umeå University, Umeå 901 87 Sweden

* Shared correspondence: [lena.gunhaga@umu.se](mailto:lena.gunhaga@umu.se) and [tamilarasan.palaniappan@umu.se](mailto:tamilarasan.palaniappan@umu.se)

**SUPPLEMENTARY FIGURES**


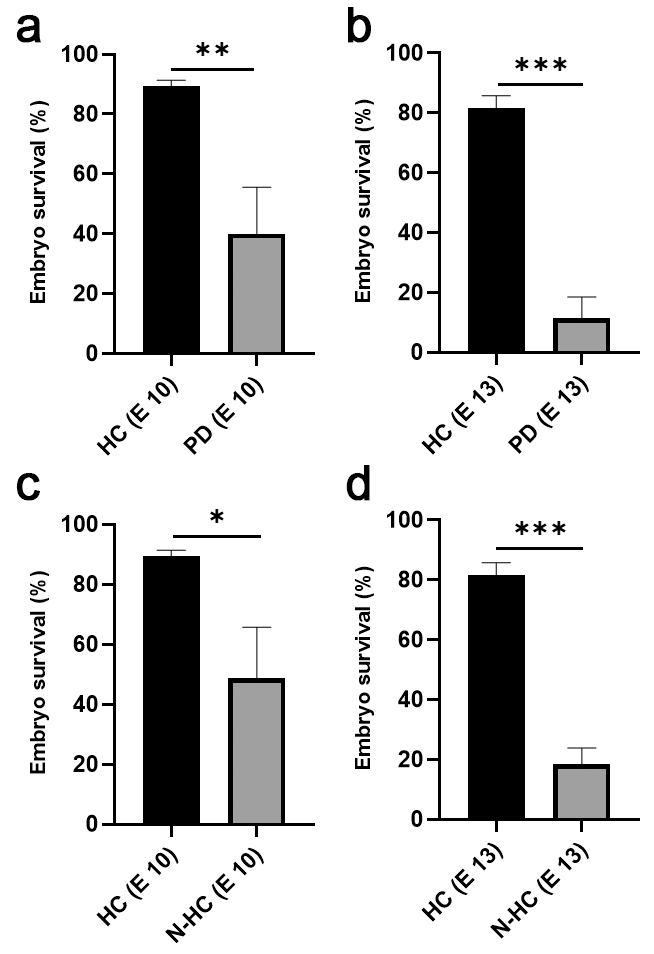


**Figure S1.** Quantification of chick embryo survival using different *in vivo*/*ex ovo* incubation methods. (a-d) The graphs indicate the survival of embryos in percentage, incubated in three different conditions: internal humidified chamber (HC), Petri dish (PD) and non-humidified chamber (N-HC).  (a,c) On Day 10 of incubation, the embryo survival was the following; HC 89±2, PD 40±16, N-HC 49±17. (b,d) On Day 13 of incubation, the embryo survival was the following; HC 81±4, PD 11±7, N-HC 18±6. The error bars indicate the standard deviation. *P<0.05; **P<0.005; ***P<0.0001.

**
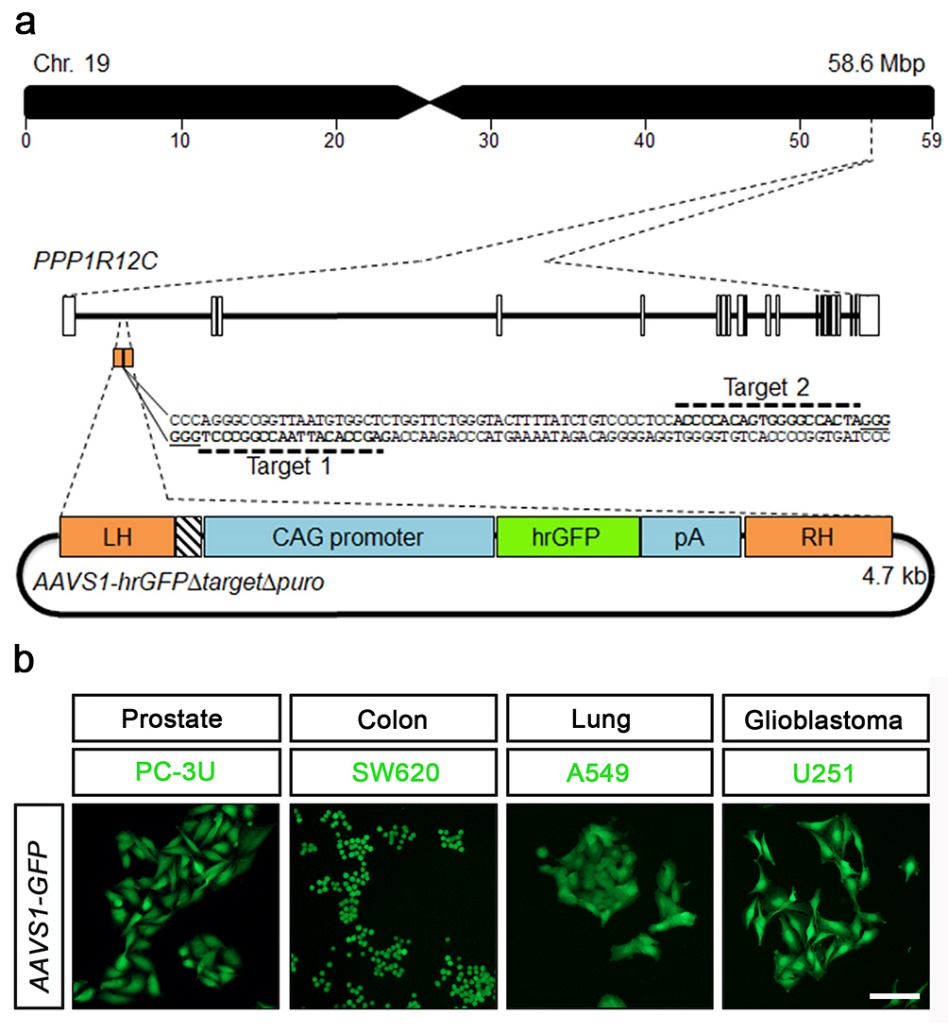
**

**Figure S2.** Generation of stable human cancer cell lines expressing GFP. Design and cloning of vectors for CRISPR/Cas9n targeting of the AAVS1 locus, and the generation of GFP-stable cancer cell lines. (a) Schematic diagram of human Chr. 19 showing the location of the AAVS1 integration site in the first intron of the *PPP1R12C* gene (located on the reverse strand from 55,117,637-55,090,918). Organization of *PPP1R12C* indicating exons (open boxes) and non-coding regions (black line), with the position of the AAVS1 integration site in intron 1 (orange box). AAVS1 sgRNA targeting region indicating the sequence of sgRNA Targets 1 and 2 (dashed line) and PAM sequences (underlined). *AAVS1-hrGFP∆target∆puro* vector indicating positions of left and right homology arms (LH; RH; orange), CAG promoter and β-globin pA (light blue) and hrGFP (green). The sgRNA targeting region deleted at the 3´ end of the LH arm is indicated as a striped box (see Methods). (b) Generated and purified PC-3U, SW620, A549 and U251 cell lines that stably express GFP. Scale bar: 100 μm (b).


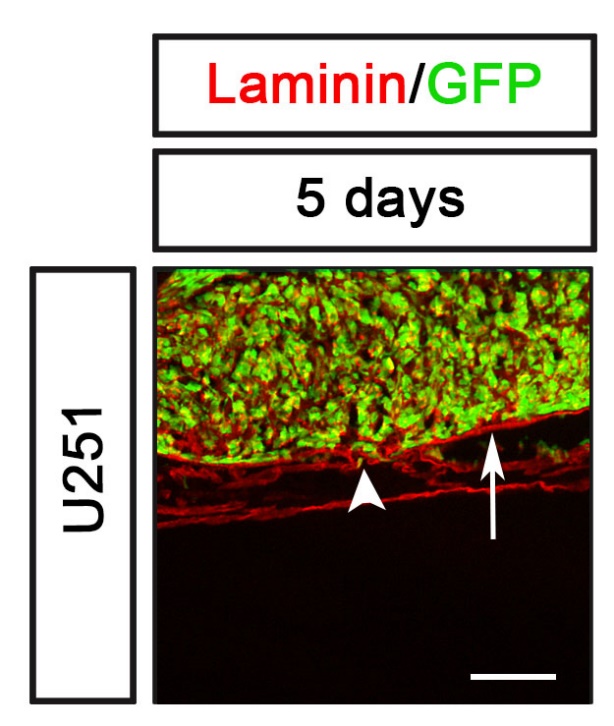


**Figure S3.** Extended culture time of U251 cells on the CAM do not result in delamination or invasion. The glioblastoma U251 cells exposed on the CAM for 5 days (from E9-E14) induced alteration of Laminin, but without clear damage of the Laminin or cancer cell invasion into the CAM, or thickening of the CAM. Arrow indicate intact laminin, and arrowhead indicate altered Laminin. Scale bar: 100 μm.

**
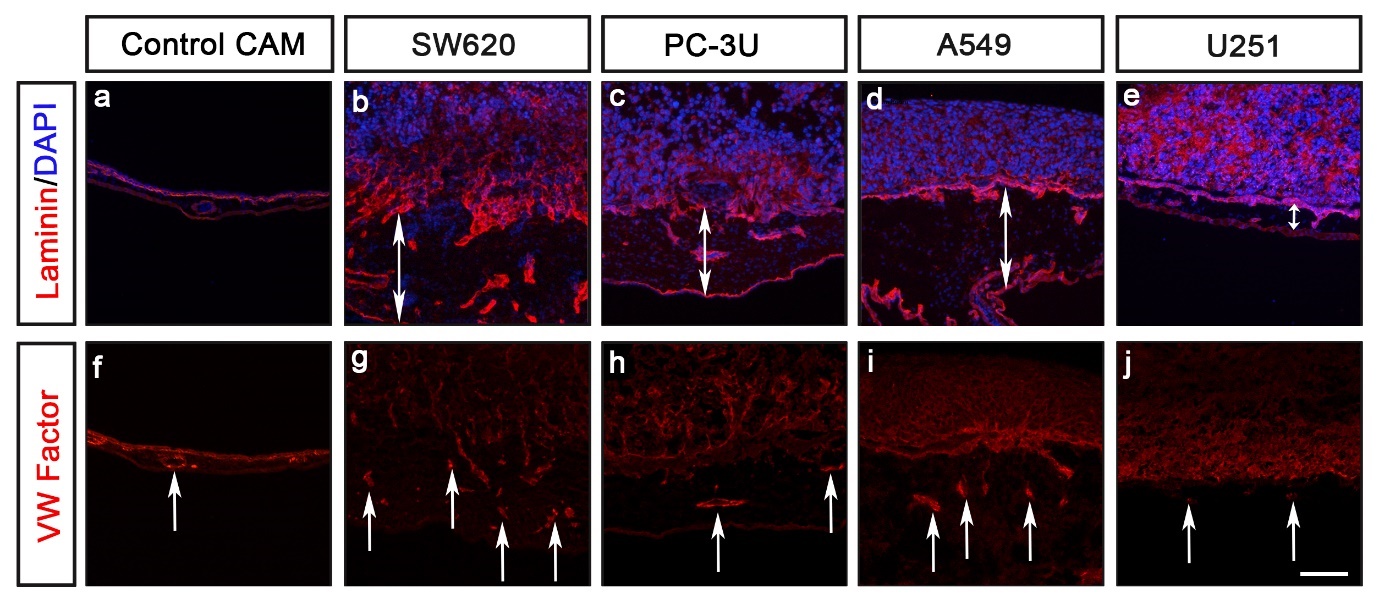
**

**Figure S4.** CAM thickening and blood vessel formation in response to metastatic cancer cells. (a-j) CAM evaluation at 1.5 d after cancer cell seeding. (a-e) CAM was visualized by anti-Laminin (red), nuclei of cells were observed by DAPI (blue). The mesenchyme was thickened in response to contact with the metastatic cancer cells SW620, PC-3U and A549 (indicated by double-arrowheads) (b, n=8; c, n=8; d, n=6). (a, e) No thickening of the mesenchyme was observed in response to the non-metastatic U251 cells (e; n=10) or control CAM without any seeded cancer cells (a; n=5). (f-j) Vessels were detected by anti-Von Willebrand Factor antigen (red). Increased blood vessel formation was observed in response to contact with the metastatic cancer cells SW620, PC-3U and A549 (g, n=4; h, n=4; i, n=4), but not with the non-metastatic U251 cells (j, n=4), or control CAM without any seeded cancer cells (f, n=4). Arrows indicate blood vessels. Scale bar: 100 μm (a-j).

**
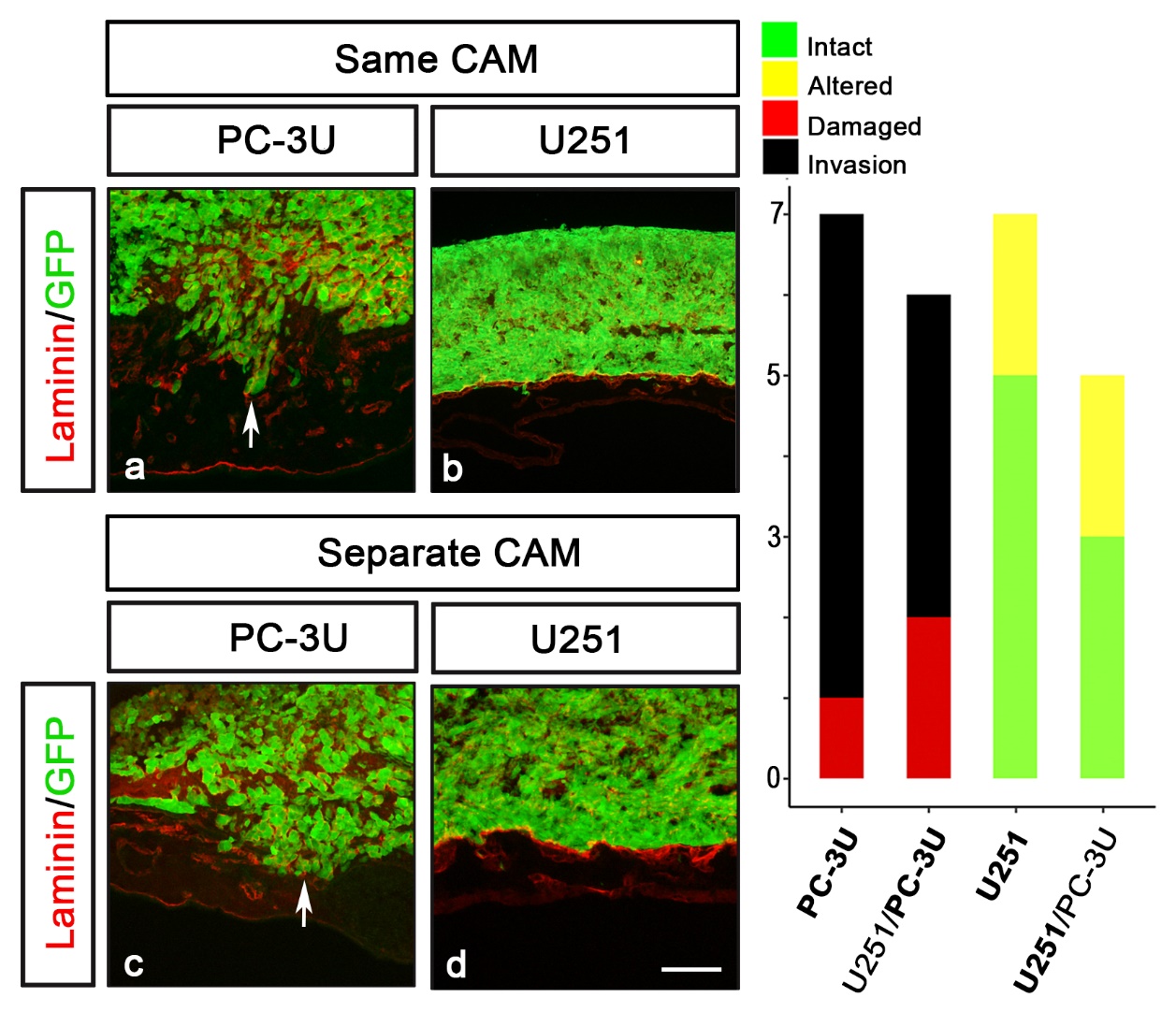
**

**Figure S5.** Different cancer cell lines assayed on the same or separate CAMs. (a-d) PC-3U and U251 GFP-expressing cells cultured on the same (a, b) or separate (c, d) CAMs gave similar CAM-Delam scoring results. (a, c) After 3.5 d of PC-3U cell seeding on the CAM, Laminin was damaged and PC-3U cells were found to be invading. (b, d) After 3.5 d of U251 cell seeding on the CAM, Laminin distribution was intact or slightly altered. The results of CAM-Delam scoring are shown in the right panel by the use of software R with package ggplot2;^1^. The y-axis indicates number of samples, and the x-axis indicates the scored cancer cell lines (in bold) on the same or separate CAM. Scale bar: 100 μm (a-d).

**
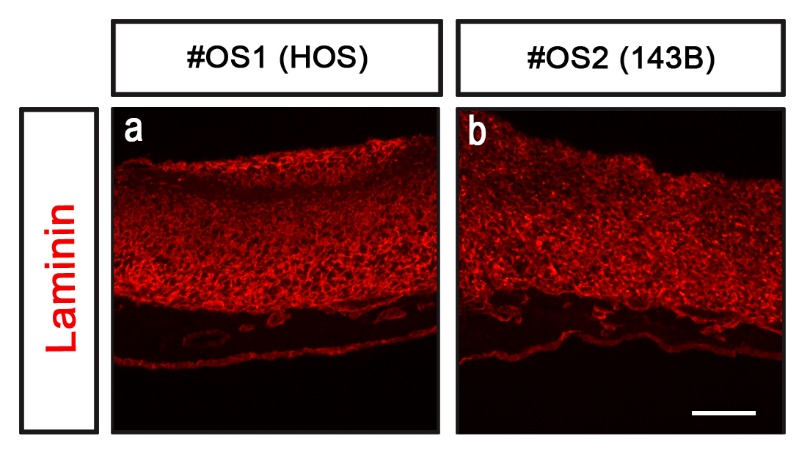
**

**Figure S6.** Osteosarcoma cells express Laminin. (a,b) The osteosarcoma #OS1 (HOS) and #OS2 (143B) cells express Laminin. Scale bar: 100 μm (a,b).


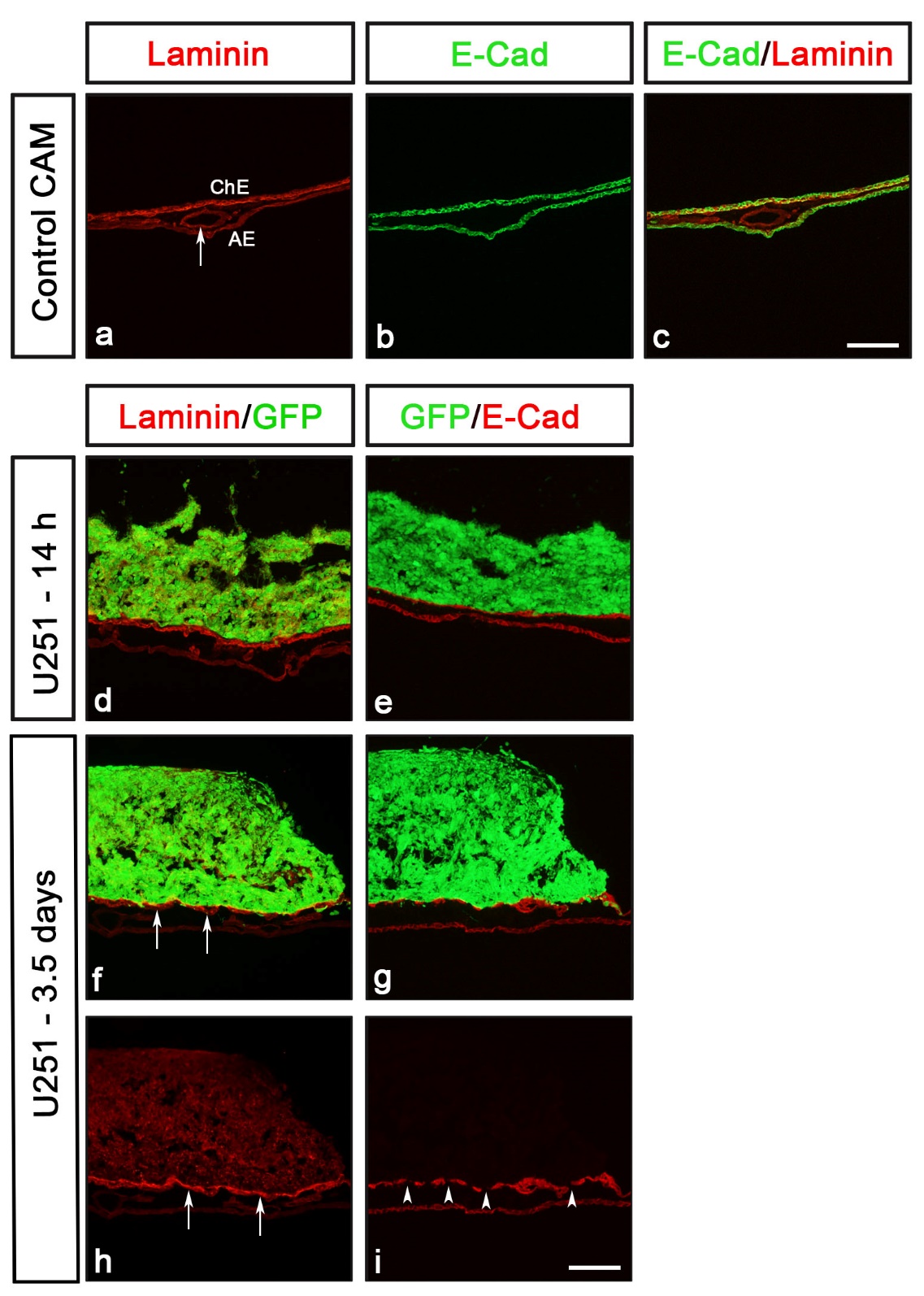


**Figure S7.** Laminin and E-cadherin expression in the CAM. (a-c) An intact CAM at 3.5 d of incubation co-stained with anti-Laminin (a, red) and E-cadherin (b, green). Laminin expression was somewhat stronger in the upper chorionic epithelium compared to the lower allantoic epithelium of the CAM. Arrow in (a) indicates a blood vessel in the CAM. (d-j) Laminin and E-Cadherin expression at 14 h and 3.5 d after U251 cell seeding, respectively. Note that the laminin layer can be intact (arrows in f, h), even when the E-cadherin epithelia is disrupted (arrowheads in i). Abbreviations: ChE – chorionic epithelium, AE – allantoic epithelium. Scale bars: 100 µm (a-c; d-i).

**SUPPLEMENTARY VIDEO CAPTIONS**

**Video S1.** Cracking fertilized eggs in a weighing boat and placing in an internally humidified chamber. On Day 3 of incubation, eggs were cracked and the yolk and its associated embryo were quickly laid into a weighing boat, which was placed in an internally humidified chamber.

**Video S2.** Seeding cancer cells inside silicon rings on the CAM. At Day 10 of incubation, the humidified chambers were opened and silicon rings were placed on the top of the CAM (indicated by arrow heads) to restrict the area for cell seeding, but never placed on the largest blood vessels. Human cancer cells were seeded on the CAM in each silicon ring, the lid of the humidified chamber was closed and incubated to a time point of interest (0.5-3.5 days).

**REFERENCES**

1 Wickham, H. ggplot2: Elegant Graphics for Data Analysis. (Springer-Verlag New York, 2016).
